# SpineCreator: a Graphical User Interface for the Creation of Layered Neural Models

**DOI:** 10.1007/s12021-016-9311-z

**Published:** 2016-09-15

**Authors:** A. J. Cope, P. Richmond, S. S. James, K. Gurney, D. J. Allerton

**Affiliations:** 1 Department of Computer Science, University of Sheffield, Sheffield, South Yorkshire UK; 2 Department of Psychology, University of Sheffield, Sheffield, South Yorkshire UK; 3 Department of Automatic and Control Systems Engineering, University of Sheffield, Sheffield, South Yorkshire UK

**Keywords:** Modelling tool, GUI, SpineML, Spiking neurons, Neuronal networks

## Abstract

There is a growing requirement in computational neuroscience for tools that permit collaborative model building, model sharing, combining existing models into a larger system (multi-scale model integration), and are able to simulate models using a variety of simulation engines and hardware platforms. Layered XML model specification formats solve many of these problems, however they are difficult to write and visualise without tools. Here we describe a new graphical software tool, SpineCreator, which facilitates the creation and visualisation of layered models of point spiking neurons or rate coded neurons without requiring the need for programming. We demonstrate the tool through the reproduction and visualisation of published models and show simulation results using code generation interfaced directly into SpineCreator. As a unique application for the graphical creation of neural networks, SpineCreator represents an important step forward for neuronal modelling.

## Introduction

### Overview

With the launch of large multidisciplinary projects, such as the Open Worm and Human Brain Project, neural models are becoming more complex and detailed. Accurate models of regions of the brain involve many types of neuron with complex patterns of synaptic connections, and models can encompass many brain regions. Such models are difficult to understand unless presented using graphical visualisation, especially for collaborators from other fields. In addition, there is a requirement for tools that permit collaborative model building, model sharing, combining existing models into a larger system (multi-scale model integration), and are able to simulate models using a variety of simulation engines and hardware platforms.

XML based model descriptions are recognised as a solution to this latter set of problems (Gleeson et al. 2010; Cannon et al. 2014; Hucka et al. 2003; Richmond et al. 2013). Importantly, they separate the model description from the simulation method, thus freeing the model from dependency on a single simulation platform. They are also declarative and machine readable (allowing efficient code-generation for simulation), portable between different hardware platforms and software, provide strict standards for model description, and allow models to be validated to ensure syntactic accuracy. These features greatly aid collaborative model building, as a set of modeling standards minimise divergence in the way models are specified; subsequently, models can be simulated using the hardware and software tools available to each collaborator. Sharing is facilitated by the interoperability of models described in a strict validatable format, as the common description format guarantees that models developed by different groups can be integrated to form larger systems. By utilising a layered approach (Raikov and De Schutter 2012), XML formats can separate semantic layers: for example, the equations used to simulate the neurons are separated from the network structure of the model, and the model separated from the experimental paradigm. This approach enables efficient reuse of computational elements in different models.

Despite these advantages, XML based models are difficult and time consuming to create by hand, and it is difficult even for a user familiar with XML to read and visualise a model directly from an XML file. This limitation is especially important in multidisciplinary projects, where it is vital that modellers can easily and clearly share ideas with collaborators from other fields. For these reasons XML formats benefit greatly from associated creation tools, which can either allow an efficient condensed specification of the model (i.e. programming language interfaces) or advanced visual manipulation of the model structure (i.e. graphical interfaces). The impact of content-authoring tools can be seen from the extensive toolsets that surround the most successful XML formats (Gleeson et al. 2010; Cannon et al. 2014; Hucka et al. 2003). Visualisation tools can take one of two forms. The first is as an extension to a model creation toolchain - once a model is described it can then be visualised as read-only. The second is a graphical creation tool, where the visualisation is used to build and interact with the model. The advantage of the second form is that it also improves the accessibility of modelling, which is often predicated upon experience in computer programming, thus allowing students and researchers with non-computational backgrounds (e.g. Psychology and Biology) to contribute to modelling. For this reason we focus on the second form of visualisation tool.

We have named our tool SpineCreator, as the XML format behind our tool is SpineML (Richmond et al. 2013), an XML format for the description of neural models. This format permits modelling at the level of networks of spiking point neurons (SNN) or rate-coded neural units (RCN). Our tool provides an easily accessible yet powerful graphical interface for model creation, and additional functionality to extend the capabilities of the toolchain (e.g. by enabling the use of neuron locations in specifying neural connectivity).

### Key Principles

#### Accessibility

Several existing model creation tools utilise programming languages to specify models (specifically Python which uses scripts (small non-compiled programs)) including PyNN (Davison et al. 2008), Brian (Goodman and Brette 2008), and pyNEST (Eppler et al. 2008)). These tools allow the efficient description of models using programming structures (e.g. for-loops), however they necessarily require familiarity with computer programming, which reduces the accessibility of both the models and the modelling approach to experimentalists, theoreticians, and students lacking experience in programming. We here advocate an approach where users enter as much information as possible in a conceptual form using graphical representations of the model, including the entry of differential equations rather than their iterative solutions, in order to promote accessibility.

#### Informative Model Presentation

Except for extremely simple models, in most current tools used for model creation the user must extract information from the model description to generate a separate diagram in order to present the model; diagrams are often used in publications rather than lists of equations to describe model architectures (Nordlie et al. 2009). The tool presented here uses the visual representation of the model in 2D and 3D as the means of *building and interacting with* the model, rather than considering it a post-hoc product of a formal description. This tight integration of model construction and visualisation greatly facilitates the design process and identification of errors in the model, for example by highlighting the afferent connections of a single neuron to check for correctly generated connectivity.

#### Facilitation of Model Creation

To simplify code generation it is sometimes helpful to limit the high level descriptive features of an XML language. One example in SpineML is the specification of connectivity between neural populations. All types of connection scheme are supported as connectivity can be described by source neuron and destination neuron connection pairs, however it may be convenient to the user to describe the connectivity in other ways. For example, the connectivity between two neural populations may be described as a function of the distance between each pair of neurons; this is not supported in SpineML, however a creation tool can remove these limitations by adding functionality to describe distance based connectivity. Models can then be output as pure declarative SpineML for simulation or sharing, with a procedural method of generating the connectivity specified in a separate file specific to the creation tool (or annotations within the XML).

#### Access to Simulators and Simulation Results

Many procedural, programming language based, modelling tools allow both support for simulating models in external simulators and the collection of data from simulations into memory. The data from the simulation can then be analysed and visualised using the tools available to the programming language, which in the case of Python are extensive. It is not possible (but also not necessary) for a graphical interface to match the level of flexibility found in programming languages, however it is necessary for the interface to provide access to simulation tools and the retrieval of data for graphical evaluation.

## Methods

### Models

We now describe briefly three neural models at differing levels of detail, from a rate-coded network to a biophysically derived microcircuit model. These models have been created using our graphical tool and are used in the following section to illustrate the capabilities and flexibility of our software. Full details of all the models can be found by downloading them from the SpineML website (http://spineml.github.io/models/) and opening them in SpineCreator.

#### Basal Ganglia RCN

**Fig. 1:**
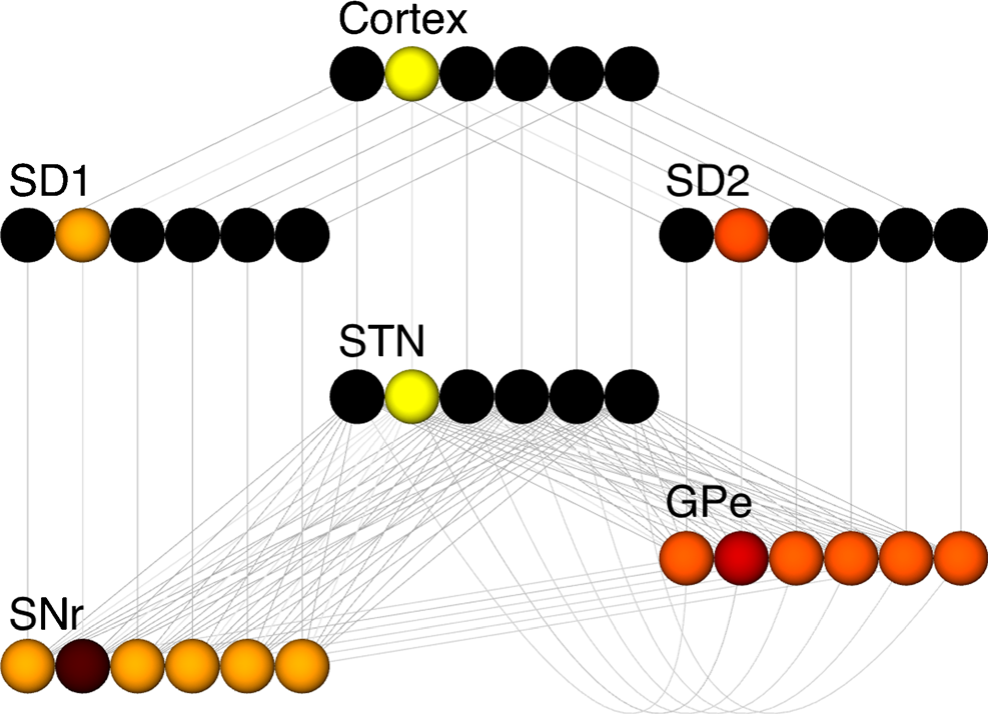
Diagram of the GPR Basal Ganglia model extracted using the network layer output feature in SpineCreator (labels added as postprocessing). *Colour* represents example activity from a simulation run in the LIN units using the ‘hot’ colourmap. Cortex: Cortical input to the model. SD1: Striatum with D1 type dopamine receptors. SD2: Striatum with D2 type dopamine receptors. STN: Subthalamic Nucleus. SNr: Substantia Nigra pars Reticulata. GPe: Globus Pallidus external segment. All connectivity is directed from top to bottom, except GPe to STN activity, which loops from the GPe to the STN

As an example of an RCN model we use a network describing action selection in the Basal Ganglia (Gurney et al. 2001a, b) (referred to hereafter as the GPR model) (Fig. 1). This model consists of rate-coded leaky integrator neurons (LINs). The model consists of a channel-based architecture, which can select between these channels by disinhibition of the selected channel in the model’s output. Further details of the model and results are available in the original paper (Gurney et al. 2001b).

#### Basal Ganglia SNN

This model is a new model based upon the GPR Basal Ganglia model described above. The GPR rate-coded model is translated into a spiking neuron model using leaky integrate and fire (LIF) models of neural dynamics. Since each LIN represents a group of spiking neurons, each is replaced by several LIF neuron models. A study of population behaviour of neurons in the Frontal Eye Fields of macaque monkeys found that robust neural responses could be obtained with seven neurons or seven repeated trials (Bichot et al. 2001), so we replace each LIN with seven LIF neuron models on the understanding that this represents a lower bound on the number of neurons that can provide a robust group response. Each channel through the BG is therefore expanded to seven LIF neurons wide. We replace the one-to-one connections between LIN units with all-to-all connections between the seven neurons in a channel and reduce all the weights in the model sevenfold as there are seven times more inputs to each LIF neuron than to each LIN unit. Next, to convert between the LIN input values and offsets and the input and intrinsic currents to the LIF neuron, all current values in the LIF model (excepting the intrinsic current in the STN) are multiplied tenfold (this value is chosen by hand to allow transmission of information between the layers). The remaining changes for the conversion consist of replacing the analog cortical input to the model with a regular spiking input, adding variation to the membrane time constants (originally all 25 ms) so that all LIN neurons do not spike simultaneously (for this the time constants were obtained from a uniform distribution with a minimum value of 20 ms and a maximum value of 30 ms), and adding exponentially decaying current based synapses with a time constant of 4 ms. In addition the model provides a better qualitative match to the results of the RCN version if the influence of dopamine on the D2 receptors is slightly reduced.

#### Striatal Model

This model is an anatomically accurate model of the striatum - the main input nucleus to the Basal Ganglia. The model comprises a biophysically based description of a 300 μm cube of the Striatum, with numbers of each neuron types and connectivity commensurate with that volume. The original model was written as a combination of a C numerical simulation and a set of Matlab scripts for generating parameters, a form not amenable to further development or sharing as the model details are obscured by the syntax. The model uses 2-variable point neurons with physiologically realistic attributes such a dopaminergic modulation (Humphries et al. 2009a) and gap junctions (Humphries et al. 2009b), based on the Izhikevich neuron model. This model will hereafter be referred to as the *Striatum Model*. Translating this model into SpineML facilitates sharing, portability, collaboration and accessibility, which are impractical in the model’s currently implemented form of Matlab and C.

The full description of this model in SpineML is difficult if written using a text editor, as the model uses a distance based connectivity scheme which must be described as a list of explicit source, destination and delay triplets (Humphries et al. 2010). We will show how SpineCreator’s extended connectivity types allow for the automated generation of this connectivity, and how SpineCreator facilitates the compact description of this complex model in SpineML.

## Results

### Overview

**Fig. 2:**
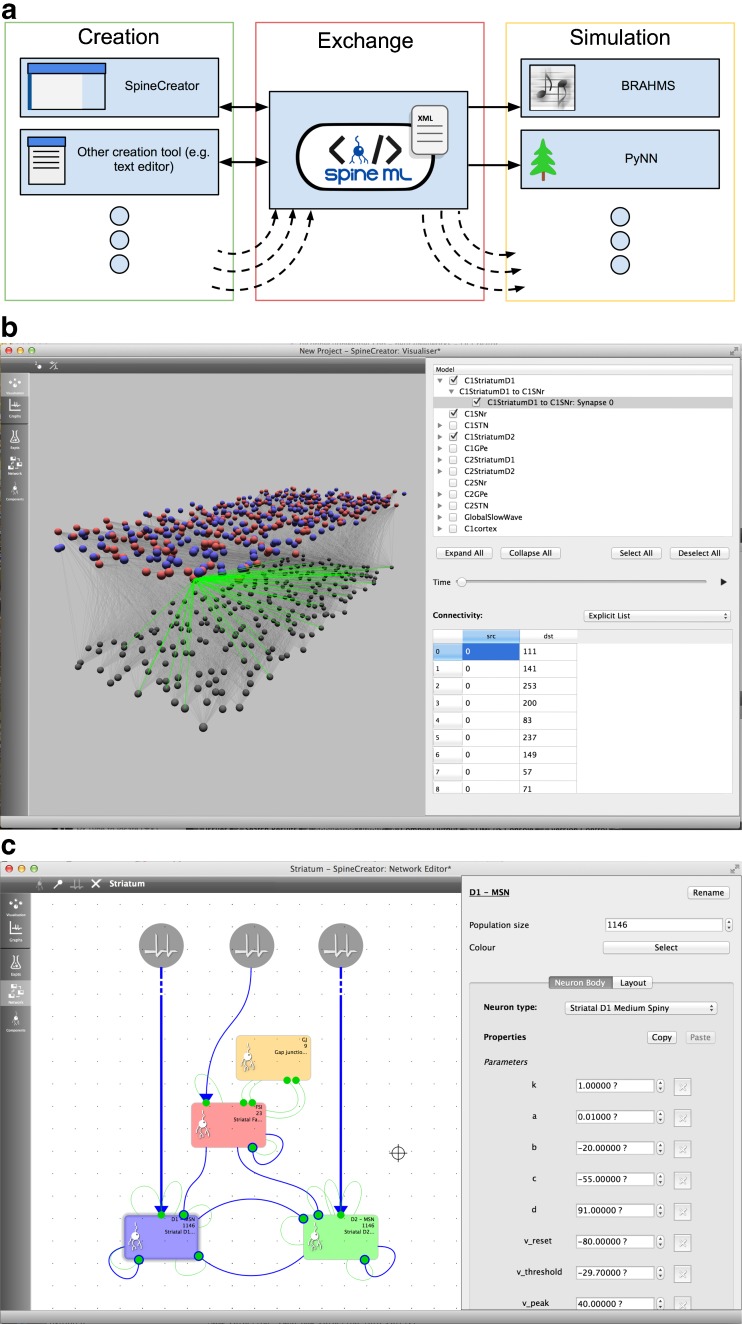
Overview of SpineCreator (**a**) SpineCreator’s role in a SpineML toolchain. SpineCreator is one of several creation tools able to exchange models through the SpineML format, and simulate models by exporting through SpineML to compatible simulators (such as the reference simulator using BRAHMS, and a range of simulators as listed on the website (http://spineml.github.io/simulators/)). (**b**) Visualisation interface showing D1-type (*red*) and D2-type (*blue*) Medium Spiny Neurons connecting to neurons in the Substantia Nigra pars reticulata (*gray*). The afferent connections for a single D1-type neuron are highlighted in *green*. (**c**) Network editor interface showing the Striatal Model

Here we present an Open Source tool for the creation of SNN and RCN models in the SpineML format based on a **Graphical User Interface** (GUI), called **SpineCreator** (SPIking NEuron/NEtwork CREATOR). The tool is written in C++ using the Qt cross platform libraries and development tools. Qt was chosen to allow the creation of an application for cross-platform deployment (Windows, OSX and Linux). SpineCreator is designed to fit into the SpineML toolchain as shown in Fig. 2↓. The source code is available online at https://github.com/SpineML/SpineCreator.

### Outline of the SpineCreator Interface

The SpineCreator GUI provides an interface with a one-to-one mapping between XML tags and the GUI interface elements. The stages of the modelling process are also analogous to the levels of description used to investigate architectures in the brain, from individual neurons to complete sensori-motor systems, followed by an external experimental paradigm. The GUI elements are implemented to allow a user to apply their existing knowledge of software UI design features such as undo/redo, recently opened file lists, drag and drop and property editing.

**Fig. 3:**
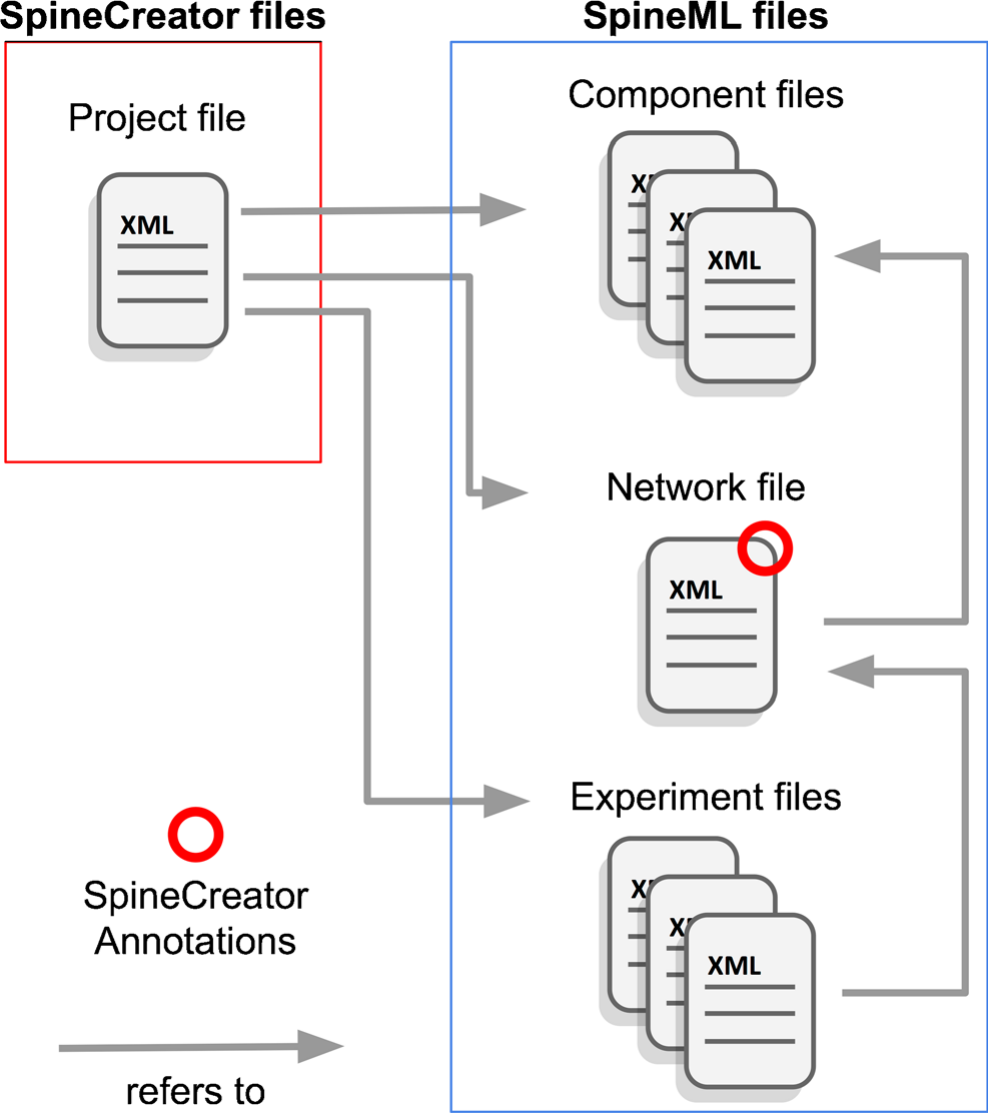
SpineCreator file structure. The Project file contains a list of all files contained in the Project. These comprise a complete SpineML model and Experiments

SpineCreator uses the ‘interface metaphor’ (a user interface function that utilises specific knowledge that users have from other domains) of a *Project* to contain a SpineML model (a single SpineML Network which references a set of SpineML Components) and a complete SpineML Experiment (or set of Experiments). Multiple Projects can be open simultaneously in SpineCreator. The elements of a Project are stored in a single working directory as individual SpineML XML files, along with an XML Project Description File that contains a list of the Project files. In addition SpineCreator stores additional information it needs to visualise the model or describe complex connectivity in XML Annotations tags within the SpineML. This allows the model to be easily edited using other software, while the XML structure of the Project and Annotations allows these elements to be easily utilised by other editing software. To facilitate this the XML format used in Project files and Annotations is described on the SpineCreator website at http://spineml.github.io/spinecreator/ (Fig. 3).

SpineCreator’s functionality consists of five main functions, each of which occupies a separate tab in the left hand bar of the main program window (see Fig. 2↑). The tabs comprise:

- **Component Creation.** SpineML Components representing neuron body dynamics, weight update rules, synaptic dynamics, or general dynamical processes, which can be created or imported, and modified using a graphical interface.
- **Network Creation.** SpineML Network files can be imported and combined, or created. SpineML Components in the same project can be used in the network.
- **Experiment Creation.** SpineML Experiments for each project can be created and modified.
- **Graphing.** Plots Analog or Event data logged from experiment runs. The plots update automatically when new experiments are run. Line and raster plots of logged data can be visualised and saved as Portable Document Format (.pdf) or Portable Network Graphics (.png) files.
- **3D visualisation.** The 3D layout of the network can be constructed and visualised. The neurons in Populations can be assigned locations in 3D space using procedural layout descriptions which can be created in SpineCreator. Connectivity patterns can be visualised, and the extended connection types that SpineCreator supports can be configured (see “Extended connectivity types↓” section). This visualisation can be output as a Portable Networks Graphic (png) or Scalable Vector Graphics (svg) image file.

We shall now demonstrate the workflow of SpineCreator for creating an SNN model using an example.

### Component Creation

**Fig. 4:**
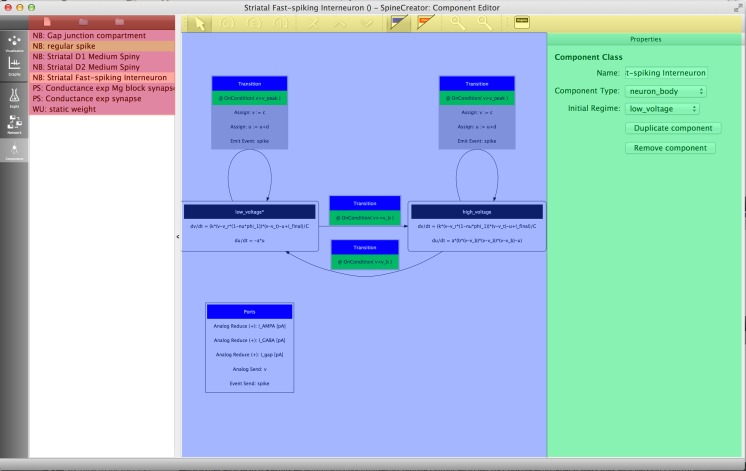
The Component Creation interface showing the FSI Component for the Striatal Model with the main UI elements highlighted by colours. *Red*: list of Components in the current Project and buttons for new/add/remove Components. *Yellow*: toolbar for adding component elements. *Blue*: diagram of the dynamics of the currently assigned Component. *Green*: context sensitive panel to modify the properties of the currently selected element in the Component

The Component Creation tab of SpineCreator is used to author SpineML Components. The interface is shown in Fig. 4↓.

The Component list provides an overview of the Components in the current Project, and is colour coded to provide immediate visual feedback to show which Components are valid (i.e. have complete definitions), invalid, and are in use in the Network, allowing the user to quickly detect errors in Components. Component dynamics are displayed graphically, as shown in Fig. 4↓, where they describe the Striatum model’s FSI neuron behaviour (replicated from Humphries et al. (2009b)). Importantly, Components can be modified, renamed and duplicated here even if they are in use. In the Striatum Model, all neuron types are extended versions of a form of the Izhikevich neuron model (Izhikevich 2007). This means that the process of creating the individual neuron types can be simplified by first creating or importing a SpineML Component for this neuron type, and then duplicating and modifying this component to form the three neuron types in the Striatal Model. By reusing an existing Izhikevich Component, the time taken to create the Components is significantly reduced, as is the likelihood of errors. Finished Components can be exported as separate SpineML files, for use in other Networks and Projects. To prevent mistakes such as deleting an element of a Component being costly in terms of development time there are separate undo stacks provided for each Component.

It should be noted that dimensionality units can be added to Parameters, State Variables and ports, and is used in the Network Creation section of the software to reduce errors by only allowing ports with matching types (event/analog/impulse) and dimensions (mV/pA etc…) to be connected.

After creation, the Components can be used to build networks in the **Network Creation** section of SpineCreator to build the model.

### Network Creation

Components created in the Component Creation section of SpineCreator, or imported from the file menu, are added to the Components available within the Project. These components can then be used in the Network Creation section of SpineCreator to construct SNN models.

Figure 2↑ (C) shows the Network Creation section of SpineCreator with the Striatal model. The left hand section of the interface shows a large 2D view of the model, topped by a toolbar allowing the addition of new objects to the network or the removal of existing objects. The right hand section of the interface shows a context sensitive panel for detailed configuration of the Network. An undo stack is also provided for the Network, allowing mistakes such as unintentional deleting of objects to be rectified easily.

Not all information about the model is presented to the user at the same time, in order to provide an uncluttered interface where useful information is not obscured by irrelevant information. The organisation of the information presented is as follows.

#### 2D Interface

This high level structural overview of the model is always present, which includes the Populations comprising the model, the Projections between them, and the Generic Inputs connecting components. These are presented in a 2D graphic which the user can arrange to form a flow diagram, as shown in Fig. 2↑. The user can therefore arrange the 2D layout using drag and drop to match the conceptual diagrams of the model; in Fig. 1↑ the correspondence between the conceptual diagram for the GPR model and the 2D Network Creation visualisation of the model can be seen. By presenting the model structure in 2D, the objects that make up the model can be arranged to clearly show their relationships to other objects, avoiding the possibility of objects obscuring each other as can occur in a 3D projected view. In addition the Populations have further information presented in this view: the Population name, the number of neurons in the Population, and the component type used by the Population. Populations can be assigned a display colour to facilitate visual identification and grouping. Projections also display extra information: thicker lines and a set of dots at the start of the projection indicate the number of Synapses in a Projection with more than one Synapse, and line colour is used to indicate the connection type used for Projections. Displaying this information ensures that the user always has an overview of the structure of the model, which can then be actively interrogated to retrieve or alter detailed information on single objects through the second level - *selection*.

Objects in the 2D graphic can be selected individually, or as a group (using the shift key to add to the selection, or the right mouse button to select objects inside a dragged box). Selecting a group of objects allows these objects to be moved together in the 2D graphic. Individual selection displays an interface with information about the selected object in the panel to the right of the 2D view. These diagrams can be exported for inclusion in manuscripts as either Portable Networks Graphic (png) or Scalable Vector Graphics (svg) image files.

#### Context Sensitive Properties Panel

The interface presented in this panel is different depending on the class of object selected, and allows the object to be configured. For example, selecting a Population object presents an interface where the name, size, colour and Component of the Population can be set. Once a Component is assigned for the Population, the input interface changes to present an interface for configuring the available Properties (StateVariables and Parameters) of the assigned Component (see Fig. 2↑). If several Populations need to share the same Properties (even if the Components differ) the copy and paste buttons can be used to copy and paste the Property configuration from one Population to another.

### 3D Visualisation

The 2D Network Creation interface provides an efficient and clear method of creating a model. However for some models, such as the Striatal Model described here, 3D information about neuron locations within a Population or connectivity between Populations is structurally important, and the 3D interface provides a more natural way to configure and observe connectivity.

#### 3D Model Visualisation Interface

3D visualisation is provided in the Visualisation pane of SpineCreator. The interface consists of a 3D viewport to the left of the interface, topped by a toolbar with options to toggle the display of neuron indices and to toggle isometric projection of the model. To the right is a panel with a tree diagram of the network for selecting objects to configure and toggling which objects are visible. Below the tree diagram is a section with a configuration interface for the currently selected object. Figure 2↑ (B) shows the Visualisation interface.

**Fig. 5:**
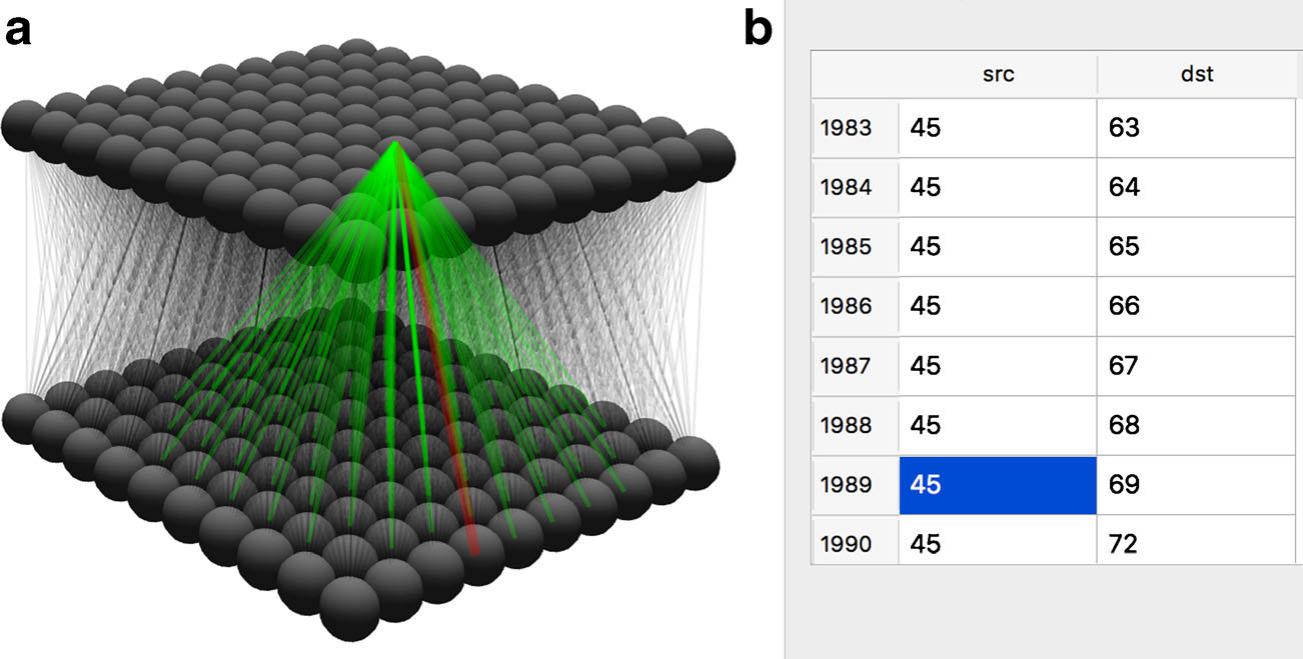
**a** An example of Projection connections from a neuron visualised in SpineCreator (currently selected connection highlighted red, other connections green, the spheres are the individual neurons). **b** Connectivity table for the same Projection as in A (only a few of the thousands of connections listed are shown). With the visual presentation all connections can be seen simultaneously, and topographic patterns are apparent, while the table only permits a subset of the connections to be viewed

The advantages of the 3D visualisation over lists is illustrated in Fig. 5↓ for an example set of connections between two populations of neurons: it is clear that the representation of connectivity for the Projection presented in A is clearer and more informative than that in B, in which only a few of several thousand connections are shown, and topographic mappings cannot be discerned. The highlighting in the 3D diagram demonstrates the context sensitive highlighting of connectivity in the 3D visualisation - connectivity can be highlighted by source neuron, destination neuron, or the individual connection. This more informative method of presentation reduces the chances of errors due to incorrect data entry or connection parameterisation through visual validation.

**Fig. 6:**
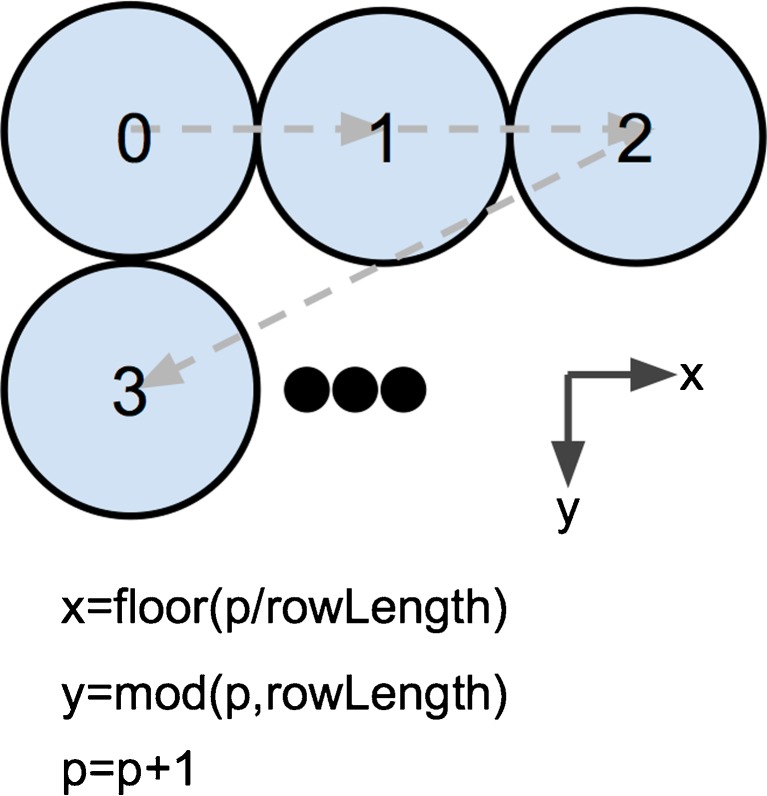
Procedural layout generation for a grid with a row length of three. *p* is increased at each iteration, and the *x* and *y* co-ordinates of that neuron are calculated using the respective formulae. The layout method can be conceptualised as an animation, with the equations determining how to transition between adjacent frames, and the index of each frame corresponding to the index of the neuron that should be placed at these co-ordinates

**Fig. 7:**
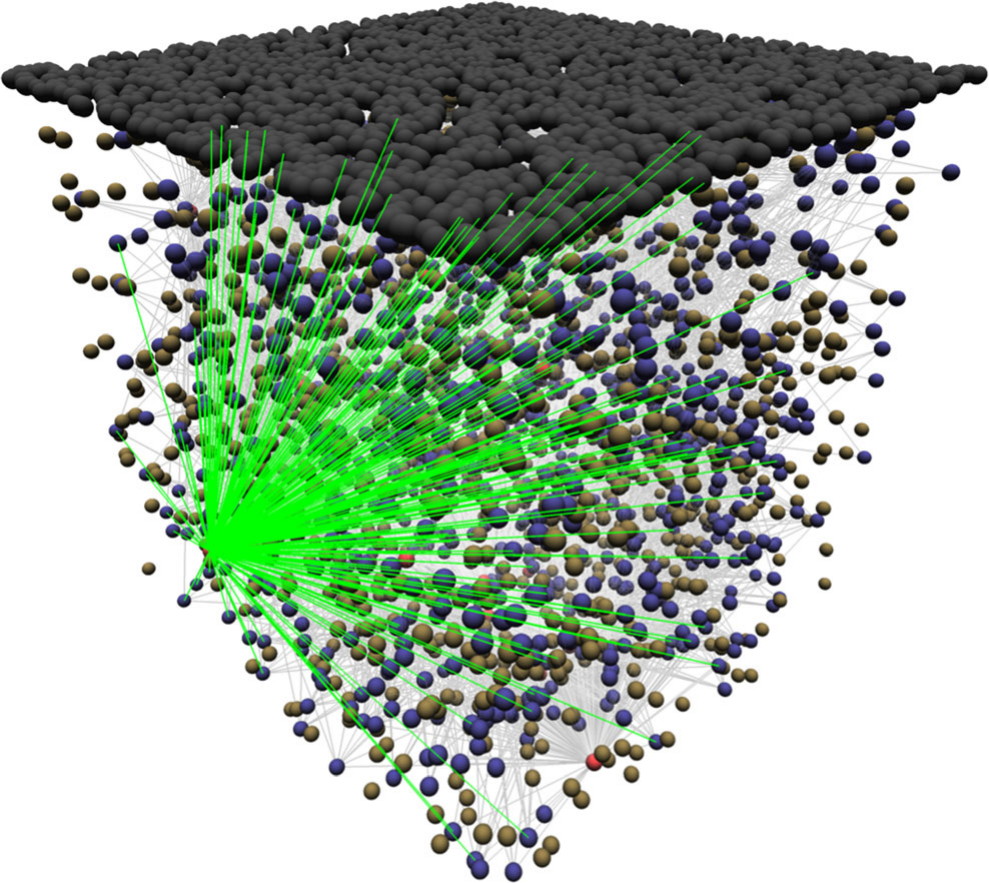
Connections (*green*) of a selected FSI in the Striatal Model to all target MSN D1s. *Grey* balls are cortical inputs, *blue* are MSN D1s, *yellow* are MSN D2s and *red* are FSIs. Connections to MSN D1s from other FSIs are *light grey*

Another example, this time with interdigitated populations, is shown in Figs. 6 and 7↓. This example shows the connectivity between one FSI and its target MSN D1s. The visualisation allows the 3D structure of the connections to be seen.

#### 3D Location Generation

To describe the 3D layout of neurons within a Population, SpineCreator provides a procedural method, which iterates through each neuron in turn and calculates the x, y and z co-ordinates using a set of user specified equations to advance values from their previous states. The process can be considered as an animation, with each subsequent frame representing the location of the subsequent indexed neuron in the population. In addition to x, y and z extra state variables can be added and updated each iteration. If a variable is used in an equation then the value from the previous iteration will be inserted (or zero for the first iteration), unless the value has been updated in the current iteration. Figure 6↓ shows a diagram of the procedural method for a grid with a row length of 3 μm. This layout was used in the BG SNN model to create six channels consisting of seven neurons each. New types of layout generation are specified using a graphical interface.

The equations used in a layout can also include uniform random numbers by using the *rand*() term. A **seed** can be specified in order to generate consistent random layouts, and a **minimum distance** can be specified to prevent neuron co-ordinates from being closer than is biologically realistic. A 3D layout, using random numbers to specify the x, y and z co-ordinates of each neuron, was used to generate the co-ordinates of the neurons in the Striatal Model. Using procedural layouts increases the speed of specifying co-ordinates in comparison with entering explicit co-ordinate lists, and the neuron co-ordinates can then be used with SpineCreator’s extended connectivity types, which will now be described.

#### Extended Connectivity Types

One advantage of a creation tool is that it can provide functionality that is not present in the underlying XML format. SpineML describes several connectivity types, however most forms of connectivity must be specified as an explicit list of [source index, destination index, delay] triplets (ensuring that any model can be supported with minimal simulator functionality). This requirement makes specifying connection types such as Gaussian receptive fields difficult, as explicit lists must be generated for the connections, with a corresponding list for the weights. To simplify this process, SpineCreator provides a means of extending the connectivity types of SpineML via Python scripts. These scripts are added to SpineCreator through the Settings/Preferences dialog, and once added appear in the list of connection types. Although the creation of an extended connectivity type requires knowledge of programming in Python, using and configuring one does not. To allow non-programmers to use and configure extended connectivity types a set of formatted comments is added to the top of the Python script: these specify parameters to pass to the script, and define how SpineCreator should provide a graphical interface for these parameters. An example script is shown below:
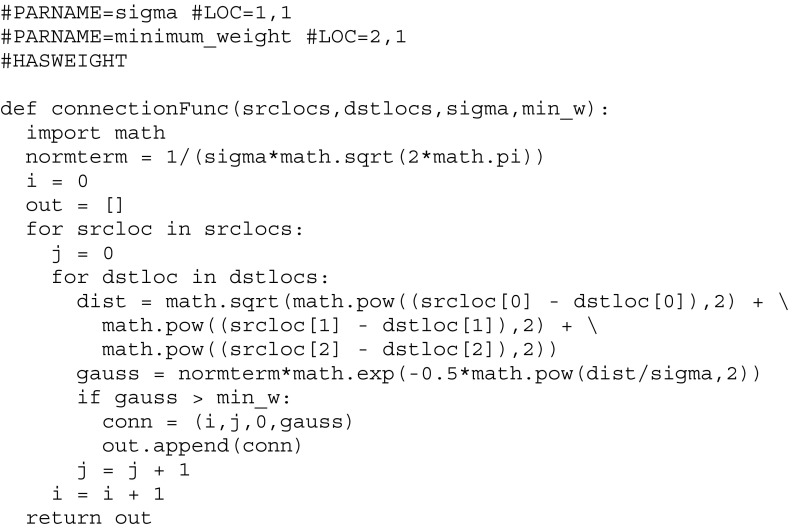


Adding parameters to the SpineCreator GUI is performed using the #PARNAME comment, which gives a name used as a label for the parameter, and a #LOC which describes the row and column where the parameter should be added in a grid for laying out the parameters. The order of the parameters in the code denotes the order they have in the corresponding Python function call, and allows the label to have a more descriptive name than the variable used in the function. In addition there are two more comments that are parsed; #HASWEIGHT and #HASDELAY, which inform SpineCreator if the script needs to generate a weight and/or a delay. If a weight is generated SpineCreator will provide a drop-down list of the corresponding Properties in the WeightUpdate Component, and the selected Property will have the weight values inserted when the connectivity is generated.

The function itself has arguments srclocs and dstlocs, which are Lists of Tuples, each Tuple containing the x, y, and z co-ordinates of the neuron at that index in the List.

Generating the explicit list of connections for larger Populations is slow, requiring a lot of computation to be performed, and therefore we minimise the amount of generation using the following strategies. Within SpineCreator the connectivity lists are not regenerated unless the script text, script parameters, or the source or destination sizes change. When writing the model out for simulation or storage, it is important that SpineCreator stores both the means of generating these extended connectivities, as well as the full ConnectionList in order that the XML version of the model is always a valid and accurate SpineML model. To achieve this, only if the connectivity has not already been generated by SpineCreator is it generated, and the full connectivity is saved in the SpineML model as a ConnectionList, and the Python script and parameter values are saved separately in the ConnectionList Annotations tag. This method has two main advantages. Firstly it guarantees an accurate SpineML model is always saved, and can therefore be loaded into other creation tools. Secondly, it speeds the process of loading a model into SpineCreator, as the connectivity does not need to be generated each time.

It should additionally be noted that Python scripting is defined in SpineCreator’s codebase as a ‘generator’ class, which acts via a ConnectionList class. This method of constructing the codebase allows that future standardised connectivity description formats can be added with minimal effort to the developers. Pragmatically, however, any future additional specification methods should still store the connectivity as a ConnectionList in the XML to maximise simulator support. Standardisation of certain Annotations within the ConnectionList tag could then provide a means of sharing the generation method between tools.

### Experiment Creation, Simulation and Simulation Data Visualisation

#### Experiment Creation

**Fig. 8:**
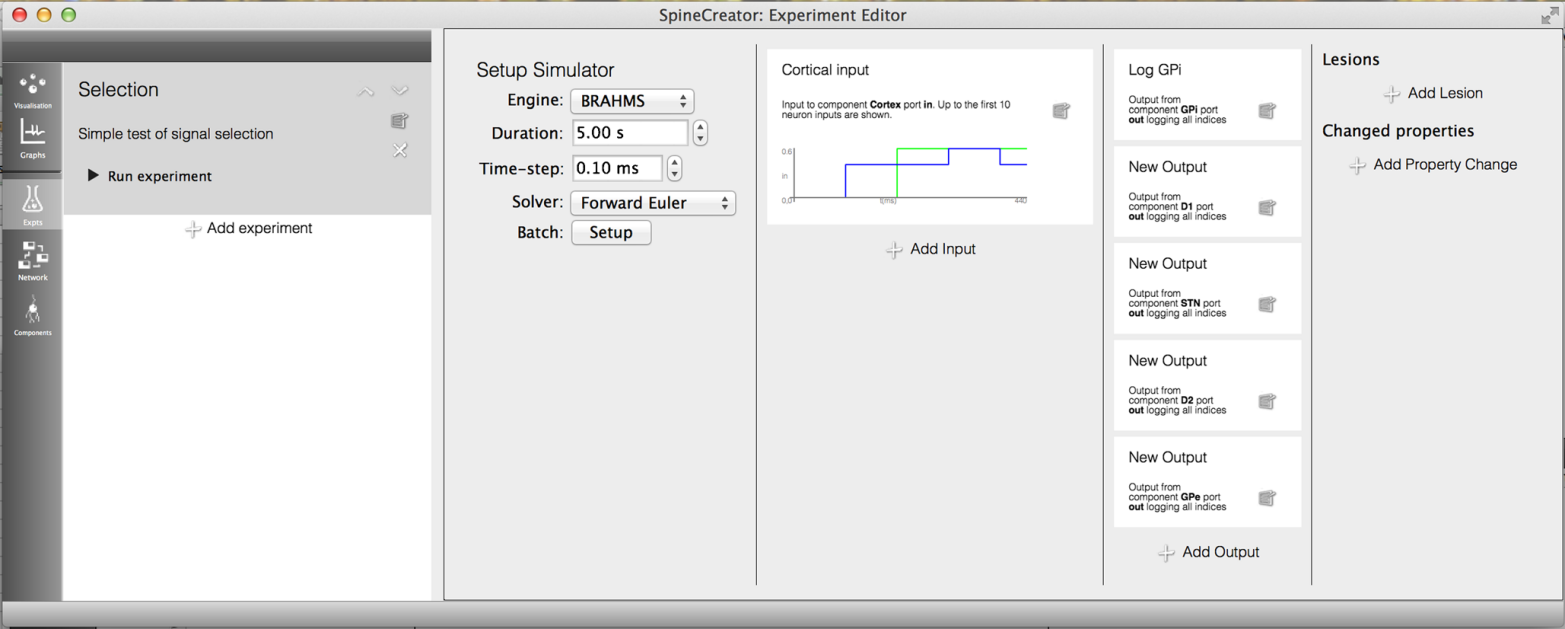
SpineCreator Experiment editor interface. A simple experiment for the GPR Basal Ganglia model is shown, with inputs, outputs and property changes

In SpineML, Experiments are separated from the model description: a feature encouraged in other model formats (Gleeson et al. 2010; Cannon et al. 2014; Raikov et al. 2011). This separation can be conceptualised as the difference between an experimental animal (the model) and the experimental paradigm (electrodes used to record data, what stimuli to present, what neuropharmacological manipulations to apply) (Waltemath et al. 2011). A single animal can be involved in a range of experiments, and therefore it is important to have a separate layer to describe the experimental procedure. SpineML Experiments can be managed in the Experiment Creation section of SpineCreator, with the ability to add experiments and specify their names and text descriptions. An example of an Experiment for the GPR model is shown in Fig. 8↓. Here a time-varying signal is added to the first two channels of the model, and the responses of several nuclei are recorded, with only the activity of the first two neurons in the D1 Striatal Population recorded. The effect of randomised values for the D1 Striatum dopamine input are investigated using a Property Change to override the value in the model. By using Property Changes, collaborators can investigate the effect of different property values without changing the Network.

The interface is based on the interface metaphor of a workbook, with the panel to the left of the interface listing the Experiment entries. When these are selected the columns to the right fill with text descriptions of the manipulations for the Experiment. By using sentences with bold fonts to emphasise key data, information about the Experiment can be found at a glance.

#### Simulation

As SpineML is a simulator independent model storage format it aims to support a wide range of simulators. As support for simulators increases it is important that SpineCreator allows easy addition of simulators by the user. Access to simulators from the graphical interface is not essential, but is required to meet SpineCreator’s of making modelling more accessible. To this end simulators are supported through scripts (for example, BASH on UNIX and Batch file on Windows) that operate on a model stored in SpineML. These scripts can be configured using an extensible list of system environment variables. Thus the user can be given a simple set of instructions by a simulator author that allows them to add support for that simulator to SpineCreator. This script based system also allows the same scripts to be used by other SpineML model creation tools or from the command line with a hand written SpineML model.

**Fig. 9:**
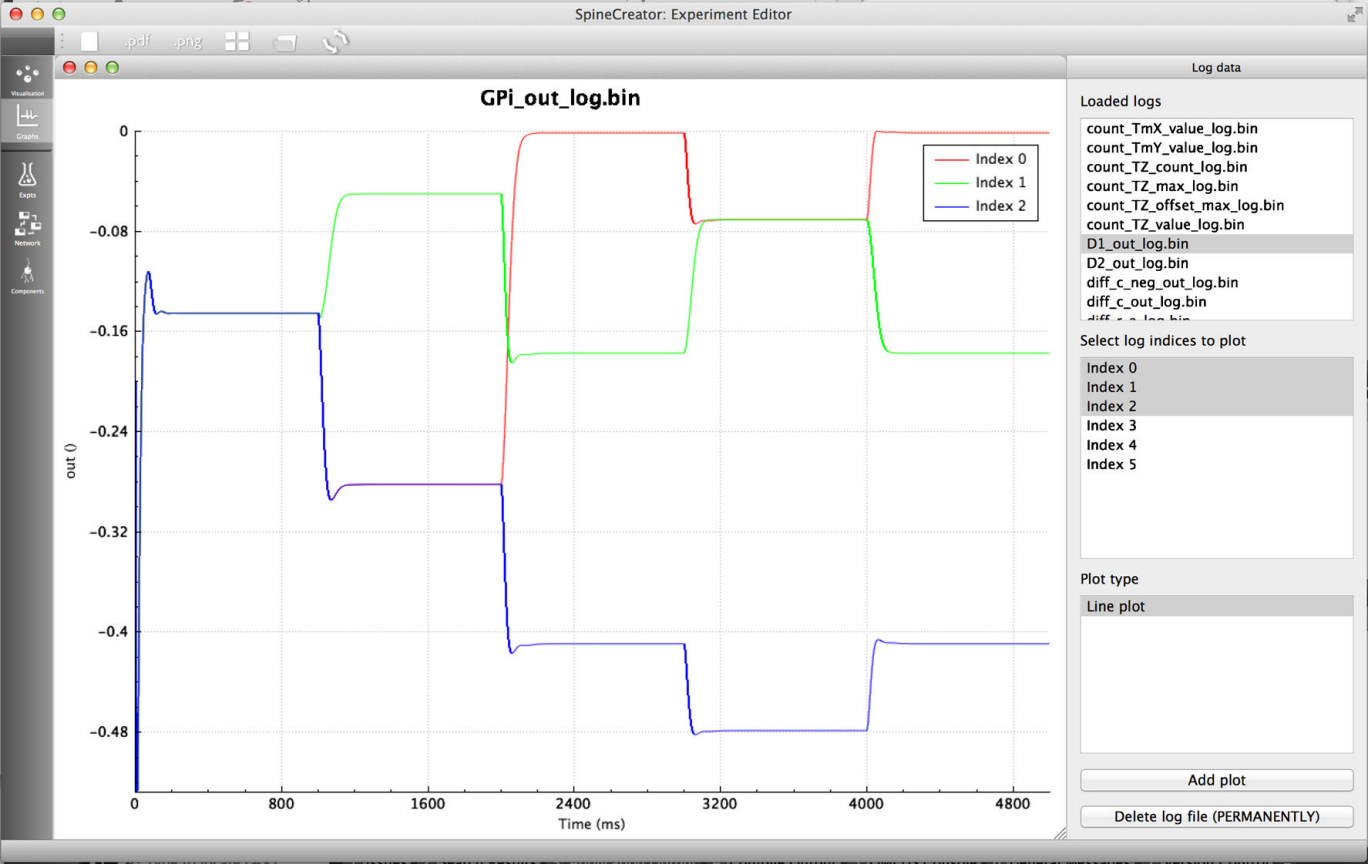
Left: 3D visualisation from SpineCreator of the GPR model (**a**) and the SNN version (**b**). *Right*: Results from the GPR model (**a**) and the SNN version (**b**). Varying signals (*dashed line*) are fed into channels one and two while the remaining channels have no signal, as represented by channel three. The output of the GPi for each channel (*solid line*) is shown. The SNN output spikes are convolved with normalised Gaussian functions (*σ* = 100 *ms*) for comparison with the rate-coded output. Selection on a channel occurs when the GPi output decreases below 0.1, or 10 Hz. Both models show the same pattern of selection, with dual selection when channels 1 and 2 have equal input values

The BRAHMS code generation target is provided as the Reference simulator, and supports all features of the SpineML language.. The results of simulation for the GPR model using BRAHMS via SpineML code generation can be seen in Fig. 9.

Other simulators are supported as described in the original SpineML paper (Richmond et al 2013), and ongoing development and support of simulator targets for SpineML is kept up to date on the SpineML website at http://spineml.github.io/simulators/, where installation instructions for the SpineML BRAHMS code generation target can be found.

#### Logging and Graphing

**Fig. 10:**
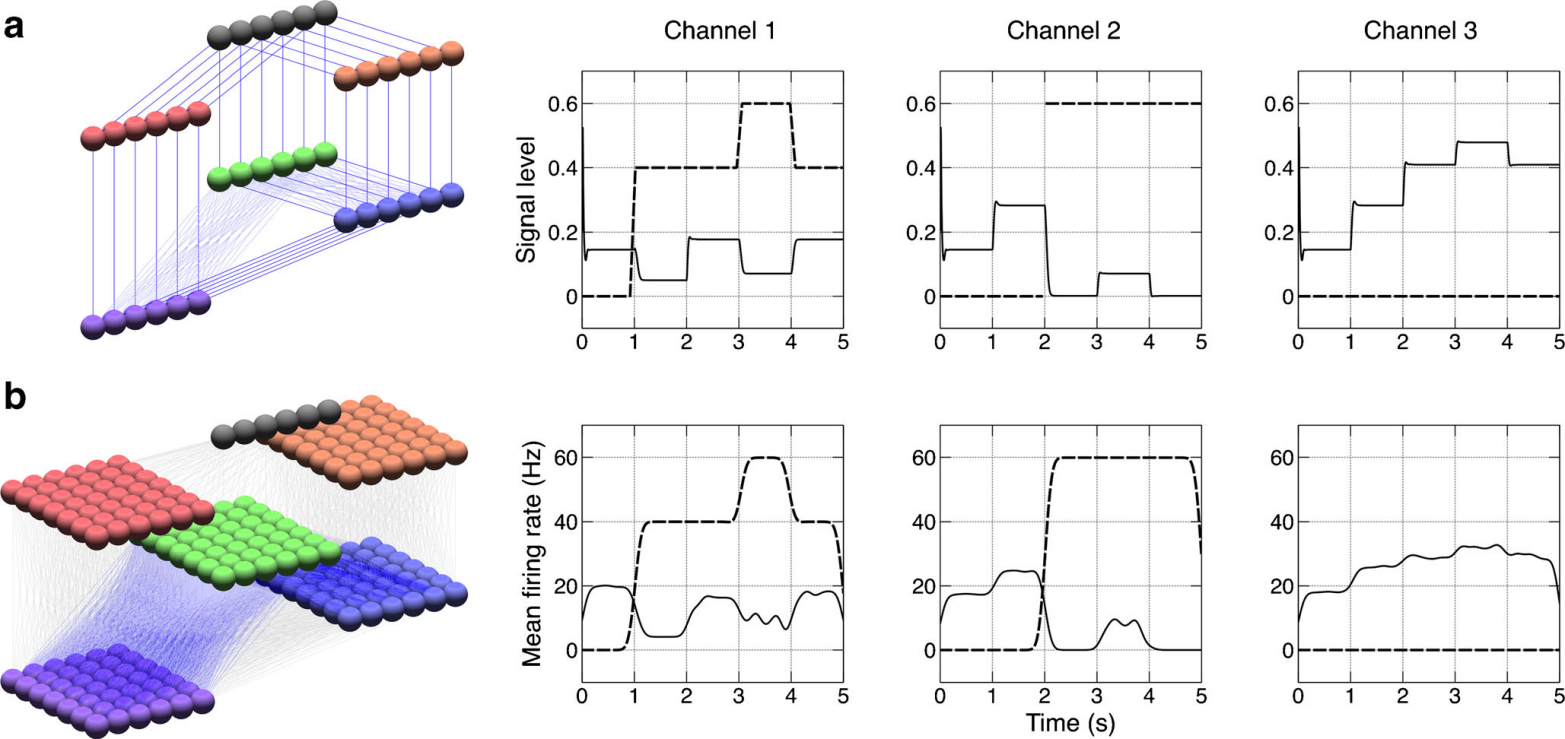
SpineCreator Graphing interface. The results of the experiment shown in Fig. 8↑ are plotted for the first three channels of the BG model

SpineCreator enables simulation data to be logged through the SpineML Experiment layer. These logs are then presented within the Graphing panel to the user, and can be plotted as line graphs for analog data and raster plots for event data. Graph drawing is undertaken using QCustomPlot (Eichhammer 2015). This facility is intended to provide immediate visualisation of the data for greater accessibility and for more rapid iteration of model development than is possible with external programs, as the process of loading logs and updating graphs between simulation runs is handled automatically by SpineCreator through communication with the simulator, while external programs are likely to require manual updating of graph plots. However, more complex analysis is possible using external programs, aided by an XML file written alongside the data file for each log that describes the source, type, and quantity of data logged. Figure 10 shows the interface while plotting the results of the experiment from “Experiment Creation, Simulation and Simulation Data Visualisation↑” section.

### Features to Facilitate Collaboration and Dissemination

#### Version Control

In any collaborative or shared model creation software, versioning is important. We therefore have integrated the Mercurial version control system (VCS) ( http://mercurial.selenic.com/) into SpineCreator using a unified versioning structure that will support additional VCS in the future. The VCS is automatically detected on the user’s computer and these options for version control are only presented if Mercurial is present. Currently repositories for SpineCreator Projects must be created outside of SpineCreator, but subsequently the repository can be managed and queried from within the tool.

#### Publication Quality Diagram Output

Currently there is no standardisation of the diagrams used in publications to describe models. A review (Nordlie et al. 2009) tried to identify themes and good practice; see Figs. 10↑ and 11↓ for diagrams of Components and Networks output from SpineCreator. There is currently only one diagram export format for Components (shared with lib9ML (Raikov et al. 2011) and compatible with LEMS (Gleeson et al. 2010; Cannon et al. 2014)) and two for Networks based on the recommendations found in the review. The support of Scalable Vector Graphics output allows vector graphics images to be created, which can then be edited and annotated.

**Fig. 11:**
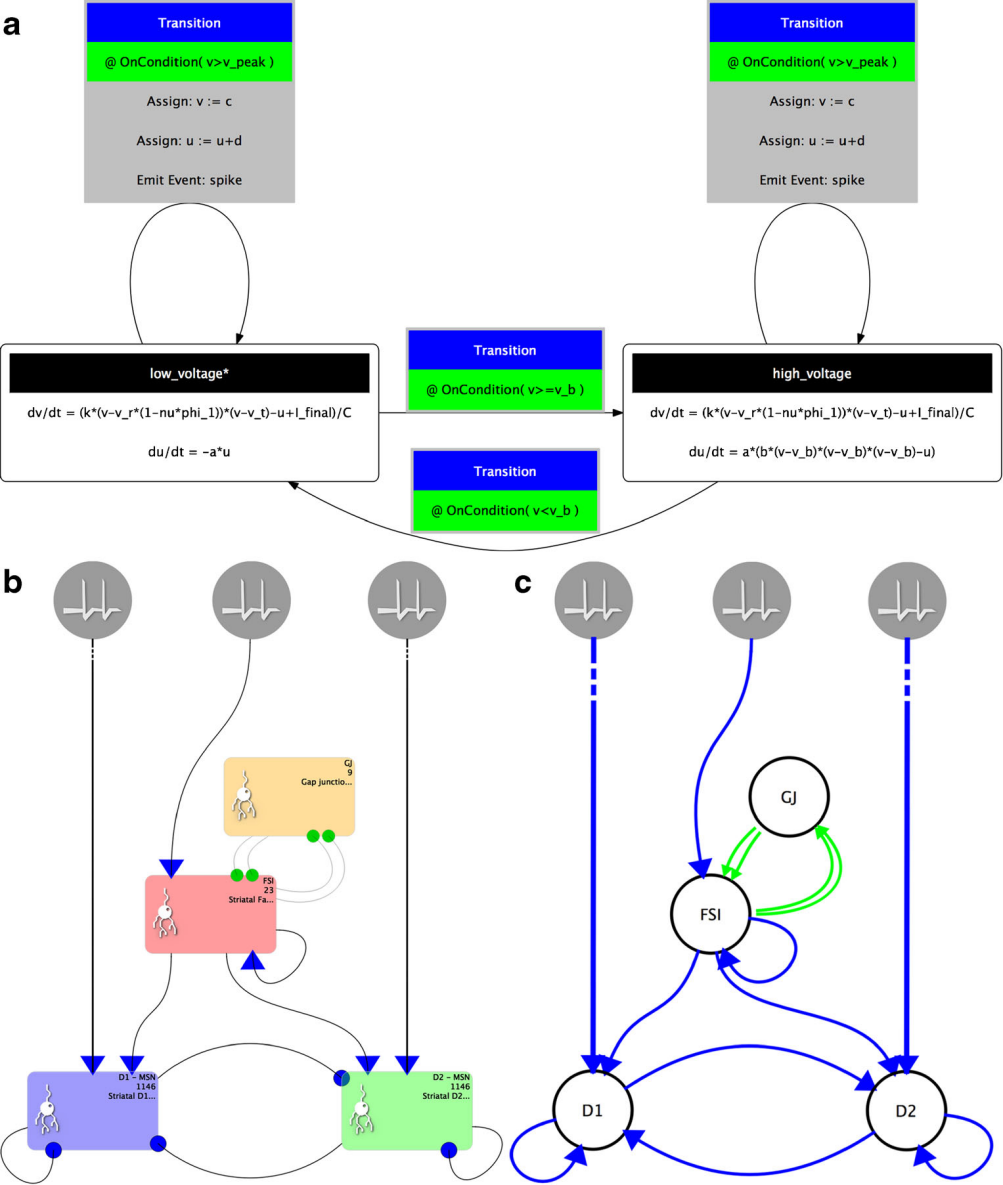
**a** a diagram of Fast-Spiking Interneuron dynamics exported by SpineCreator. **b** and **c** A diagram of Striatal microcircuit exported by SpineCreator with two different styles, one with less and one with more information density

## Discussion

In this paper we have presented SpineCreator, a GUI for the creation of neural models using the declarative XML format, SpineML. Creation tools are essential for the uptake and usability of any XML based neural model specification format, as the effort of writing models by hand in such formats is a major drawback for all but the simplest models. This interface facilitates the creation of SpineML models, and increases the accessibility of the SpineML modelling toolchain to those without a background in computer programming. The extended connectivity types allow the creation of complex rule based connection schemes, thereby extending the functionality of the SpineML format. The visualisation features of the interface allow the structure of the model to be easily presented and understood. SpineCreator imposes no limits on the size of models, barring the availability of memory on the host machine.

### Facilitating the Creation of Models

Primarily, SpineCreator is designed to facilitate the creation of neural models. This can be undertaken using SpineCreator as the sole model creation tool, however since SpineCreator saves the model in the SpineML XML exchange format models can be easily authored using several tools. For example, it may be beneficial to create repetitive structures in a programming language, where a for-loop can be used to iterate over each element. The resulting model can then be imported into SpineCreator and details can be added once the model is organised in a 2D flow-diagram form where it is easier to see the relationships between the elements. To benefit such collaboration between creation tools we provide an online resource describing the structure of the SpineCreator Project file as well as the Annotations that SpineCreator adds to the SpineML Network file. This can be found at http://spineml.github.io/spinecreator/.

Model creation is also facilitated through some of the features provided by the SpineML format structure, as using a layered approach to describe models (Raikov and De Schutter 2012) facilitates reuse. ***Components*** can be reused within the same model, and this is an approach adopted in many simulator independent and simulator dependent tools (e.g. PyNN). ***Networks*** can also be reused by their inclusion in new Projects, thus a *Systems Integration* approach, where existing models of different parts of brain function can be combined into a model of a larger part of the brain, is supported by SpineCreator.

Several further features of SpineCreator also play a role in facilitating model creation:

By only presenting information when an object is selected, and providing a clear visual indication of the selected object, SpineCreator minimises the amount of detailed information presented to the user and facilitates the identification of the information the user is seeking. This *interrogative* approach to finding information is used in SpineCreator as an alternative to presenting all information at the same time or in a specific order as in a script. This approach increases the speed with which information can be located and changed by minimising the number of options presented to the user at each stage - increasing the ease with which a model can be created and preventing overwhelming inexperienced users with a cluttered interface.

Version control is essential to any large, collaborative project. While version control is possible with any tool, as VCSs are capable of versioning even binary files, the advantage of SpineCreator is that the version control is integrated into the graphical interface. This methodology ensures that the functionality is accessible without requiring knowledge of specific command line tools. Using version control reduces the chance of model divergence, and allows verification that the right version of the model is being used to reproduce results.

The integration of standardised diagram output in SpineCreator helps model authors by reducing the time spent creating diagrams of models, and removes the possibility of inaccurate diagrams by creating the diagrams directly from the model itself. Most importantly, providing diagram output in a standard form ensures that diagrams are clear and understandable.

The extended connection types added to SpineML by SpineCreator allow complex connectivity patterns to be described simply using the graphical interface and visualised in 3D. This extension removes the requirement to use additional software or programming to create such connectivity and thus makes the SpineML toolchain more accessible to non-programmers. Likewise, the built in graphing allows simple raster and line plots of simulation outputs to be created without the use of external tools, providing immediate feedback on the results of a simulation, without having to use external graphing programs.

### Improving Accessibility Through a Clear Interface to the Model

Collaborative modelling and sharing models to promote reuse is becoming increasingly important as our understanding of neural systems increases, and models grow in scale and complexity. Large projects involving multiple disciplines, including experimentalists and theoreticians, are widespread. In such projects it is important that collaborators without a programming background can understand and contribute to computational modelling. Collaboration and sharing also increases the number of checks the model undergoes (decreasing the possibility of errors). It is also important that the end products of these projects are proliferated into the wider scientific community in a form that is both accessible and understandable. The graphical representations of models that SpineCreator provides can help with these goals.

When sharing and exchanging models it is important that the model recipient is able to understand the model rapidly. Graphical representations provide a conceptual overview of the structure of the model, and can reference the flow of the model through flow-chart style diagrams of Populations and Projections, or reference the anatomical basis of the model using three dimensional representations. Through these references the recipient of the model can more easily understand the model. This graphical representation allows SpineCreator to fit into the workflow of a large project by providing an entry point into understanding a complex model, both for modellers looking to extend or modify the model, or for experimenters looking to understand the modelling work and use it to guide further experiments. Thus, collaboration between experimentalists, theoreticians and modellers is facilitated by making the model accessible and understandable to those without a background in computer programming.

The accessibility provided by a graphical representation also enables SpineCreator to be used as an educational tool for the teaching of neuron dynamics in disciplines where computer programming skills are not taught. Currently in other common model description formats (Davison et al. 2008; Eppler et al. 2008) programming is a requirement for creating neuron dynamics whereas SpineCreator removes this requirement, making the field more accessible.

### Levels of Use

While SpineCreator can provide a clear interface for non-programmers, it also supports different levels of use depending on the user’s level of understanding. At the simplest level users can build either individual neurons as components to integrate into an existing network, or build networks out of existing components, or modify the parameters of an existing model and through experimentation discover how this affects the model’s behaviour. This process requires no programming knowledge.

Users who are unfamiliar with computer programming can use SpineCreator to build components and networks using extended Python connectivity provided for them, also without requiring any programming knowledge.

Finally, advanced users who have programming experience can create extended Python connectivities using all the features of SpineCreator.

By facilitating exporting publication quality diagrams SpineCreator can also help the dissemination of finished models for users in the research community, and can facilitate collaborative work using the version control interface built into SpineCreator.

By including this tiered approach users can utilise SpineCreator at all levels from student through to researchers, without needing to appreciate the more advanced features SpineCreator offers more experienced users.

### Comparison with Existing Tools

There is no direct comparison for SpineCreator amongst the existing model creation tools. NeuroConstruct (Gleeson et al. 2007) is a simulator independent model creation GUI utilising an XML description format for storage, however it is focused on the creation of detailed compartmental neural models and networks in the NeuroML language - for creating SNN models it has a high learning curve and unintuitive interface, and it cannot be used to construct RCN models. Aside from SpineCreator, there is no equivalent of NeuroConstruct for SNN and RCN creation.

SpikeNET (Delorme et al. 1999; Delorme and Thorpe 2003), XNBC (Vibert et al. 1997, 2003), and SNNAP (Ziv et al. 1994) are software packages that consist of an integrated creation tool and simulator. These tools focus on providing a set of standard neuron models, such as the LIF and Izhikevich models, and do not permit the user to modify the dynamical equations of one of these types, or add a new type. One requirement of the SpineML toolchain is that it can be used to create models that currently can only be created in C and Matlab, and thus these tools do not fulfill that requirement.

NeuralSyns (Sousa and Aguiar 2014) is a set of graphical tools for creating and simulating neural networks of spiking neurons. The tools focus on removing the requirement for programming, and providing 3D visualisations of neurons and simulation activity, however programming is required to add neuron model types. These requirements highlight two main differences in philosophy from SpineCreator, as NeuralSyns uses an internal format for storing models, which is not designed to work as part of a wider toolchain. This format relies on the use of a single simulator, while the SpineML format used by SpineCreator provides a full specification of the model, which allows any user to translate the XML description into standardised running code. NeuralSyns also provides graphical visualisation as a simulator feature, rather than as a part of the modelling process, instead using a graphical interface of buttons and menus. Therefore while there is some overlap between the tools there is also a clear distinction.

Several tools use the high level Python programming language for model creation (PyNN (Davison et al. 2008), Brian (Goodman and Brette 2008), pyNEST (Eppler et al. 2008)). These tools use a scripting approach where a set of functions are used to build the model. The scripting approach allows for extremely compact descriptions of models, but trades off model comprehensibility, as scripted models can be extremely difficult to read and understand. On the other hand, declarative formats are inflexible, and can require considerable duplicated content. SpineCreator tries to combine these two approaches, providing structure and constraint by using a declarative XML format to save models, but also providing tools such as copy/paste, one click duplication of Components, and extended connectivity types to facilitate the creation of these declarative models. Always, the declarative output of the procedural syntax can be visualised to confirm that the declarative model output is as expected.

### Limitations and Future Development

SpineCreator is intended as a first creation tool for the SpineML toolchain, and it is expected that it will sit alongside other creation tools, all exporting models to a shared XML format. In addition, SpineML is a proposal for the completion of the NineML specification, and as such it is the intention to integrate SpineML with NineML and NeuroML 2.0’s LEMS format.

## Information Sharing Statement

The example models presented within this paper (namely the GPR Basal Ganglia models and Striatal model) are available in complete form from the SpineML website (http://spineml.github.io). Additional documentation is also available to aid users in reproducing the experiments and results presented.

### Installation

SpineCreator is available to download from the SpineML website at http://spineml.github.io/spinecreator/, where extensive instructions are provided on installation under Macintosh OSX and Linux. There is additionally a list of supported backend simulators on the SpineML website at http://spineml.github.io/simulators/.
